# Fungal necrotizing external otitis: diagnosis, management and outcomes of 15 cases

**DOI:** 10.11604/pamj.2022.42.306.27168

**Published:** 2022-08-24

**Authors:** Amel El Korbi, Jihene Houas, Naourez Kolsi, Rachida Bouatay, Mehdi Ferjaoui, Adnene Toumi, Khaled Harrathi, Jamel Koubaa

**Affiliations:** 1 Ear Nose and Throat Department, Fattouma Bourguiba University Hospital, Monastir, Tunisia,; 2 Research Unity Interventional Radiology (LR18SP08), University of Monastir, Monastir, Tunisia,; 3 Department of Infectious Diseases, Fattouma Bourguiba University Hospital, Monastir, Tunisia

**Keywords:** External otitis, fungal, diabetes, CT-scan

## Abstract

Fungal necrotizing external otitis (NEO) is a rare disease. It is an aggressive and potentially fatal infection. The most commonly reported pathogen is Candida. We aim through this study to share our experience in the management of fungal necrotizing external otitis and discuss its diagnosis tools, anti-fungal treatment choice, and outcomes. We included fifteen patients with diagnosis criteria of fungal NEO; clinical features of NEO with positive culture swabs and/or positive serologic test to a fungal pathogen. The mean age was of 70 years with a prevalence of males. The main symptoms were otalgia (n=15) and otorrhea (n=7). Facial palsy was observed in four cases. Fungal pathogens were Candida(n=10) and Aspergillus (n=5). Complications were observed in eight cases: extension to the temporo-mandibular (n=4), abscess in the retropharyngeal space (n=2), abscess in the parapharyngeal space (n=1) and thrombophlebitis of the internal jugular vein (n=1). Six patients were treated with fluconazole, eight with voriconazole, and one patient with itraconazole. After a mean duration of 52 days of antifungal therapy, fourteen patients have been cured with normalization of the ear symptoms, biological, and imaging features. One patient died of septic shock. No recurrence of the disease was observed after a follow-up of 12 months in all cases.

## Introduction

Necrotizing external otitis (NEO) is an aggressive and potentially fatal infection, which has spread outside the confines of the external auditory canal to involve temporal bone, mastoid air cells and peri aural soft tissues [1]. Chandler first described it as a clinical entity in 1968 [1]. This entity occurs especially in elderly diabetic and immunocompromised patients with acquired immunodeficiency syndrome (AIDS) and acute leukemia [1,2].

The incidence of NEO has increased in recent times. This is can be explained by the increase in awareness of the disease and better diagnosing modalities. The most commonly reported pathogen in necrotizing otitis externa is *Pseudomonas aeruginosa* observed in 90% of cases. Fungi are rarely involved in NOE. The most common pathogen is *Aspergillus fumigatus* [2]. We report in this study a large number of patients with fungal necrotizing otitis and propose an algorithm detailing antimicrobial prescription.

## Methods

A retrospective chart review was performed in the Department of Otolaryngology Head and Neck Surgery of Fattouma Bourguiba University Hospital between 2004 and 2016. Sixty-seven patients with a diagnosis of NEO were identified in the medical records within this period. All data were collected in charts: age, gender, clinical features, imaging findings (computed tomography (CT) scan and magnetic resonance imaging (MRI)), biological results, microbiologic analysis results, treatments received, and clinical issues. We include all cases with diagnosis following criteria of fungal NEO admitted in our department, which associate: absence of clinical response, and the worsening of symptoms with intravenous anti-pseudomonas therapy in patients with the diagnosis of NEO; presence of fungi on culture swabs and/or fungal positive serologic test.

## Results

**Epidemiological and clinical presentation:** fungal NOE was diagnosed in fifteen patients within the period of study, which represents 22.4% of whole NEO cases. The mean age was of 70 years (range 54-89 years) with a prevalence of males (sex ratio=2). All patients were diabetic with an average diagnosis duration of 8 years. Two patients had chronic renal failure. Diabetic control worsened with the onset of invasive external otitis in all cases. All patients have received oral and local antibiotics before hospitalization. Quinolones were the molecules prescribed in all cases. Symptoms were made of otalgia in all patients, otorrhea in seven patients, and hearing loss in five patients. Headache and temporo-mandibular joint pain were reported in four cases. Fever was observed in three patients. Two patients noted ipsilateral facial palsy.

Stenosis of the external auditory canal was observed in 11 patients; thus tympanic membrane was seen in only four cases. Granulation tissue was present in ten patients. It was located posteriorly in the auditory canal at the junction of the bony and cartilaginous parts. We observed swelling of temporomandibular in four cases and of pinnae in two cases (Table 1). Histopathologic examination of the scrapings from the external auditory canal (EAC) did not show miceti. Biological investigations have shown a hyperglycemia with rates ranged between 1.8 and 4.8 g/L and high erythrocyte sedimentation rate (ESR) which is ranged between 33 and 110 mm/H1 (mean, 69 mm/H1).

**Table 1:** patients' demographic characteristics and clinical presentation

Demographic characteristics	n
Male	10
Female	5
Age (mean in years)	70
**Signs/symptoms**	**n**
Otalgia	15
Auricular discharge	7
Decreased hearing	5
Headache	4
Temporo-mandibular joint swelling	4
Fever	3
Facial nerve palsy	2
EAC stenosis	11
Granulation tissue	10

EAC: external auditory canal

**Microbiology and positive diagnosis:** the diagnosis was based on clinical and radiological features, microbiology, and serology results. Microbiologic cultures have shown fungi in twelve swabs. A *Candida* in seven cases and *Aspergillus* in five. In three cases, we did not yield any growth (Table 2). Serologic tests were positive for *Aspergillus* in two cases and *Candida* in six cases. They were negative in the other cases (Table 2).

**Table 2:** summary of patients' pathogens, treatment, and outcome

Case n°	Age (years)	Gender	Pathogen		Antifungal therapy	Adverse effect of antifungal therapy	Duration of antifungal	Outcome
			Culture result	Serology result				
1	89	Male	*Candida tropicalis*	+ (*Candida*)	Fluconazole	-	6 weeks	Cured
2	54	Male	*Candida albicans*	+ (*Candida*)	Fluconazole	-	4 weeks	Cured
3	61	Female	*Candida parapsilosis*	Negative	Fluconazole	-	5 weeks	Cured
4	74	Female	*Candida albicans*	Negative	Fluconazole	-	1 week	Died
5	73	Male	Negative	+ (*Candida*)	Fluconazole	-	3 weeks	Cured
6	62	Male	Negative	+ (*Candida*)	Fluconazole	-	4 weeks	Cured
7	78	Male	Negative	+ (*Candida*)	Fluconazole then itraconazole then voriconazole	Acute renal failure with hypokalemia	9 weeks	Cured
8	59	Female	*Candida albicans*	+ (*Candida*)	Fluconazole then voriconazole	-	6 weeks	Cured
9	76	Male	*Aspergillus fumigatus*	Negative	Voriconazole	-	12 weeks	Cured
10	69	Female	*Aspergillus flavus*	Negative	Voriconazole	-	12 weeks	Cured
11	64	Male	*Candida albicans*	Negative	Voriconazole	-	4 weeks	Cured
12	71	Female	*Candida albicans*	Negative	Voriconazole	-	9 weeks	Cured
13	72	Male	*Aspergillus fumigatus*	Negative	Voriconazole	-	12 weeks	Cured
14	79	Male	*Aspergillus fumigatus*	+ (*Aspergillus*)	Voriconazole	-	12 weeks	Cured
15	69	Male	*Aspergillus fumigatus*	+ (*Aspergillus*)	Voriconazole	-	14 weeks	Cured

**Figure 1:**
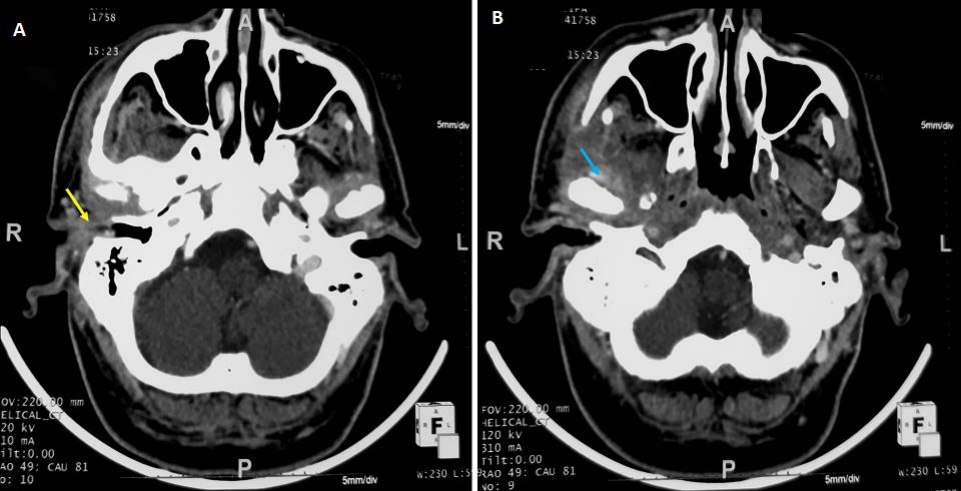
(A,B) temporal bone CT scan of a patient with right fungal NEO (yellow arrow) showing an extension of the inflammatory phenomenon to the right temporo-mandibular joint (blue arrow)

**Figure 2:**
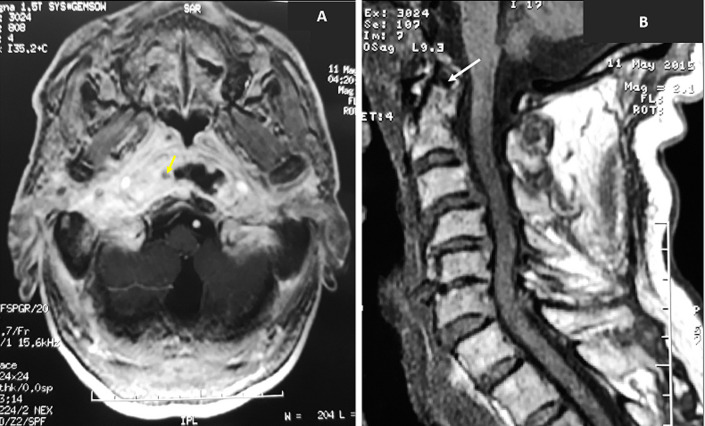
(A,B) temporal bone MRI of a patient with a right fungal NEO showing an extension laterally an abscess of the right parapharyngeal space (yellow arrow) and posteriorly to the atlo-axoidien joint (white arrow)

Computed tomography has shown bone erosion in all cases, which occurred in the tympanal bone in eleven patients, and in the cortical mastoid in four patients. An extension to the middle ear occurred in all cases. Osteolysis of facial canal was observed in two cases and temporo-mandibular joint in four cases (Figure 1). Magnetic resonance imaging (MRI) was performed in four cases with parapharyngeal involvement. It has shown an abscess in three cases (Figure 2) with an extension to the vertebral space and arthritis of the atlo-axoidien joint and thrombophlebitis of the internal jugular vein in one case.

**Treatment:** patients have primary received intravenous anti-pseudomonas treatment. We observed a lack of clinical response and worsening of symptoms in all cases. Antifungal therapy was started an average of two weeks after the diagnosis of NEO (Table 2). Eight patients have initially received fluconazole and seven patients have received voriconazole. In two cases of non-improvement with fluconazole, we have switched to voriconazole in one case and itraconazole in the other one. Acute renal failure with hypokalemia was observed in one patient treated with itraconazole. We were obliged to switch with voriconazole with favorable outcomes. The mean duration of antifungal therapy was of 52 days (7-98 days). All patients have received topical treatment with antibiotics and antifungals. Local treatment with cleaning and calibrating of the ear canal was conducted daily in all patients. None of the patients has received hyperbaric oxygen therapy as adjuvant treatment.

**Outcomes and follow-up:** the mean hospitalization duration was of 34 days (7-50 days). We observed a regression of symptoms in thirteen patients. Two patients developed facial palsy during hospitalization. One patient died of septic shock (Table 2). We have not observed a recurrence of the disease within a follow-up of 12 months. However, facial palsy did not improve in all cases.

## Discussion

Our study reports courts are among the largest series of fungal NEO in the literature; in fact, NEO is a rapidly spreading infection, which originates at the bony cartilaginous junction of the external auditory canal. It is attributed to infection after trivial trauma, such as that of aural irrigation or ear pricking [3]. We observe an increase in the incidence of fungal NEO, this could be explained by an increase in local and general antibiotic prescription without microbiology proof in patients with immunity failure with local tissue microangiopathy and even altered cerumen biochemistry [4]. Diabetes mellitus (DM) is one of the most tightly associated characteristics seen in NEO patients. It is documented that diabetes causes endarteritis and microangiopathy, leading to poor microcirculation and impaired polymorphonuclear cell function [5]. Although diabetes appears to predispose to both pseudomonal and fungal NEO, it is much more common in the former. The strong association between fungal infections and hematological malignancies and human immunodeficiency virus (HIV) is confirmed [6].

**Clinical presentation:** diagnosis is made upon clinical, microbiological, and radiological grounds and requires a high index of suspicion. There are no universally accepted specific criteria [2]. All our patients presented with classical features of NEO namely the occurrence of persistent severe otalgia with few systemic symptoms, purulent otorrhea with granulations, and resistance to local therapy for at least 10 days. Complications such as facial palsy and extension to temporo-mandibular joint seem to be more frequent in fungal NEO strengthening the aggressiveness of such disease [2]. Sterile swabs and cultures for bacterial and fungal are necessary to identify the pathogen. In our study, there was no growth in 20% of cases (3 cases). Previous antibiotics given to patients may explain negative results.

**Microbiology and positive diagnosis:** bacteria are the major cause of external otitis involvement. The most common one is *Pseudomonas aeruginosa*. Fungal NEO was until a decade ago extremely rare [7]. In the immunosuppressed, non-diabetic patients with NEO, fungi are relatively frequently isolated, especially in those with AIDS or acute leukemia. However, fungal NEO is extremely rare overall. *Aspergillus Niger, Aspergillus fumigatus*, and *Candida* species are the common fungi reported [6,8-10]. *Pseudoallescheria apiosperma, Malassezia sympodialis* and *Scedosporium apiospermum* have been also reported to involve in NEO [11-13]. The diagnosis of fungal MEO should be based on histopathologic confirmation on deep tissue biopsy or isolation from blood cultures or fistula exudates [2].

**Imaging:** high-resolution CT scan and/or MRI of the temporal bone are a useful for diagnosis. They are also useful for disease progression. CT scan allows exact analysis of bone erosion, but MRI is better in analyzing infratemporal or skull base disease involvement [8,14]. MRI does not use radiation and provides the most anatomically detailed information about the disease extent and soft tissue involvement including meninges and parotid area [15]. CT scan showed an extension to the middle ear in all our patients which seems to be an imaging diagnosis argument of such disease. However, no evident base proof exists in the literature. Other imaging investigations such as Tc^99^methyl diphosphonate bone scanning, Ga^76^citrate scanning, and Ga^67^single photon emission computed tomography (SPECT) could be associated with the couple CT-MR for diagnosis and follow-up [2].

**Treatment:** during the past thirty years, the treatment methods of NEO have changed. Treatment involves a multidisciplinary approach, with treatment planned and discussed with the specialist concerned. Successful management of NEO frequently requires collaboration with endocrinologists, radiologists, and infectious disease specialists. Aggressive and adequate control of diabetes, the correction of electrolyte imbalance must be stated at the earliest as possible. The most commonly used antifungal agent was amphotericin B. This drug has been effective in the treatment of *Aspergillus* NEO, but its substantial renal toxicity especially in patients with serious comorbidities [16]. Fluconazole and itraconazole have also been used in cases reported in the literature [6]. Voriconazole is currently recommended as a first-line treatment in cases of invasive aspergillosis and its use is increasing since 2002 [16,17]. Regarding its favorable bone penetration, tolerance, and efficacy, voriconazole is an attractive first-line therapeutic option for *Aspergillus* NEO.

Hyperbaric oxygen (HBO) is an efficient inhibitor of fungi growth, but it was used in a few cases of fungal NEO in the literature thus we cannot assess the exact value of this therapy in that entity [18,19]. Some authors suggest that surgical debridement is particularly indicated in the fungal form of NEO [2,17]. All of our patients have received a conservative treatment based on anti-fungal therapy with favorable issues in all cases. Our decision on anti-fungal prescription was based on swabs and/or serology positivity. No surgery was performed even in complicated cases.

**Outcome/survival:** complications occurring seem to be more frequent in fungal forms of NEO, which leads obviously to a higher risk of mortality. Thus, we thought that early diagnosis and anti-fungal therapy starting could decrease the mortality rate of fungal NEO. Mion *et al*. through their systemic review concluded that the absence of facial palsy, *Aspergillus* as a causative pathogen, and the absence of imaging findings were correlated with a better outcome [2].

**Limitations:** we cannot make strong conclusions through this study because of its retrospective character with no statistical analysis.

## Conclusion

Fungal NEO is a serious life-threatening infection of the external ear and skull base condition. We demonstrated through this paper that fungal NEO diagnosis and anti-fungal therapy beginning could be achieved on swabs and/or serology tests positivity avoiding thus deep biopsies under general anesthesia. We also emphasized the efficacy of voriconazole, as a first-line treatment in *Aspergillus* NOE.

### What is known about this topic

- Fungal form of NEO is more aggressive and lead to more cranial complication than the bacterial form;
- Candida and aspergillus are the two main pathogens of fungal NEO;
- Pathogens should be isolated to start anti-fungal therapy.

### What this study adds

- An extension to the middle ear was constant in our study;
- Fungal NEO diagnosis and anti-fungal therapy beginning could be achieved on swabs and/or serology tests positivity avoiding thus deep biopsies under general anesthesia.
